# Fatigue evaluated using the 16-item Parkinson Fatigue Scale (PFS-16) predicts Parkinson’s disease prognosis

**DOI:** 10.20407/fmj.2018-014

**Published:** 2019-02-06

**Authors:** Yoshiki Niimi, Sayuri Shima, Yasuaki Mizutani, Akihiro Ueda, Shinji Ito, Tatsuro Mutoh

**Affiliations:** Department of Neurology, Fujita Health University School of Medicine, Toyoake, Aichi, Japan

**Keywords:** Parkinson’s disease, Fatigue, 16-item Parkinson fatigue scale

## Abstract

**Background:**

Although fatigue is an important nonmotor symptom in Parkinson’s Disease (PD) patients, little is known about the pathophysiological details of fatigue in PD, and it is still unknown whether fatigue correlates with PD prognosis. In this study, we investigated whether fatigue in PD correlates with clinical manifestations, treatment, or patient prognosis.

**Methods:**

We recruited 75 idiopathic PD patients and used the Parkinson Fatigue Scale (PFS-16) to investigate fatigue. We compared PFS-16 scores with clinical details such as age, disease duration, daily levodopa equivalent dosage, and Hoehn & Yahr (H&Y) disease stage in the 56 patients who fully completed the questionnaire.

**Results:**

In total, 62% of subjects suffered from fatigue, as defined by a mean PFS-16 score above 3.3. There was no correlation between PFS-16 scores and disease duration or levodopa equivalent daily dose. However, there was a significant correlation between mean PFS-16 scores and a worsening grade of H&Y staging. The comparison between patients who showed stable H&Y grades (*n*=26) and patients with severely aggravated H&Y grades (*n*=7) revealed that the most significant differences were in questions 14 and 16 in the PFS-16 (*p*<0.001).

**Conclusion:**

Fatigue is common in PD patients, as demonstrated in the present study. The PFS-16 questionnaire may be helpful to predict disease prognosis.

## Introduction

Parkinson’s disease (PD) is the second most common neurodegenerative disease,^1^ and the prevalence of PD is increasing with the aging world population. Fatigue is one of the most common and disabling nonmotor symptoms in PD. Its prevalence is widely reported, ranging from 33% to 77.6%.^2,3^ Although the detailed mechanism of fatigue remains to be elucidated, fatigue occurs in all stages of PD and is a leading cause of disability in PD patients.^4^

The 16-item Parkinson Fatigue Scale (PFS-16) is a useful self-rated assessment tool to assess fatigue and its impact on daily function in PD patients. The PFS-16 includes 16 questions that reflect the symptoms of fatigue, focusing on the physical aspects and excluding emotional or cognitive elements because the latter two may occur independently in PD patients.^5^

Stocchi et al., using the PFS-16 questionnaire in Italian patients, revealed that fatigue was associated with higher clinical ratings of severity, poorer quality of life (QOL), worse social and psychological behaviors, a higher severity of depressive symptoms, and an increased prevalence of sleep disorders.^6^ Another study, this time in Japan, also reported an association between fatigue and reduced QOL.^7^ However, it is still unknown whether high fatigue scores correlate with disease progression. In this study, we focused on fatigue in PD patients and investigated whether the Japanese version of PFS-16 is useful for predicting PD prognosis.^7,8^

## Methods

### Patients

We enrolled 75 consecutive patients with idiopathic PD who visited the Fujita Health University Hospital from July 2015 to October 2015. Patients were diagnosed using the UK Parkinson’s Disease Society Brain Bank clinical diagnostic criteria.^9^ Nineteen cases were excluded because PFS-16 answers were incomplete. We followed up the remaining 56 patients until June, 2018. We defined disease duration as the period from disease onset to the registration date in 2015. The follow-up period is defined as the period from each patient’s registration date to their last follow-up examination in 2018. Of the 56 total subjects, 6 patients died during the study, and 11 patients could not attend the outpatient clinic during the follow-up period because of increased physical disability. In such cases, we adopted the latest clinical data (Table 1).

### Study variables

We investigated PFS-16 scores, clinical characteristics, and levodopa equivalent daily dose (LEDD) at recruitment. We also performed a modified Hoehn & Yahr (H&Y) staging^9^ at recruitment, and followed up H&Y staging in 2018 or at the last visit.

### Hoehn & Yahr staging

H&Y staging is designed to describe severity of PD. It provides a general estimate of clinical function combining both functional deficits (disability) and objective signs (impairment). Each scale of H&Y staging represents the disease stage as follows: 1.0: unilateral involvement only; 1.5: unilateral and axial involvement; 2.0: bilateral involvement without impairment of balance; 2.5: mild bilateral disease with recovery on pull test; 3.0: mild to moderate bilateral disease, some postural instability, physically independent; 4.0: severe disability, still able to walk or stand unassisted; 5.0: wheelchair bound or bedridden unless aided.^10^

### The 16-item Parkinson Fatigue Scale

The PFS-16 is the original questionnaire that was developed by Brown et al. in 2005 to measure fatigue severity in PD.^5^ In this assessment, responses to 16 questions are established as “strongly disagree”, “disagree”, “do not agree or disagree”, “agree”, and “strongly agree”, which are scored as 1 to 5, respectively. Previous studies have reported the validation of the Japanese version of PFS-16 for the assessment of fatigue in patients with PD.^7,8^ Table 1 shows each question in the questionnaire. We used the mean score of the 16 different questions to determine the presence of fatigue; a patient was deemed to have fatigue if the mean PFS-16 score was over 3.3, according to a previous report.^5^ We then used the mean PFS-16 score to investigate its correlation with clinical data.

### Patient groups

We divided patients into two groups according to changes in their H&Y stage results over time. One group was the H&Y stable group, which consisted of patients with no H&Y stage change during the follow-up period. The other group was the H&Y aggravated group, which consisted of subjects who exhibited a worsening of their H&Y stage by two points or more. We adopted this division according to a previous study by Zhao et al., who reported that, based on 695 patients with PD, the transition time from H&Y stages 1 to 2, 3 to 4, and 4 to 5 takes about 2 years.^11^

### Ethics

This study was approved by the Ethics Review committee of Fujita Health University, and all subjects and/or their immediate relatives provided informed written consent before participation (approval number HM15-182).

### Statistical analysis

The open-source software environment for statistical computing and graphics, R, was used for statistical analyses (https://www.r-project.org/). A significant difference was defined as *p*<0.05. Pearson’s chi-squared test was used for comparisons of age and sex. Spearman’s rank correlation coefficient was used to investigate correlation among ordinal data. The Brunner–Munzel test was used for intergroup comparisons.^12^

## Results

The clinical characteristics of the 56 enrolled patients are summarized in Table 2. During the follow-up period, 26 patients showed no change in H&Y stage, while seven patients had a severe worsening of H&Y stage (at least 2 points) compared with their stage at recruitment. The other 23 patients demonstrated a decrease in H&Y stage of between 1 and 1.5 points. There was a positive correlation between age and PFS-16 score, initial and follow-up H&Y stage and mean PFS-16 score, and H&Y worsening and PFS-16 score. However, there was no correlation between PFS-16 score and disease duration or LEDD. Of the 56 subjects in total, 36 patients (62%) had a mean PFS-16 score of above 3.3. The results of correlation coefficients between H&Y aggravation during the follow-up period and PFS-16 scores are shown in Table 1. There was a positive correlation between H&Y aggravation and PFS-16 scores for questions 2, 5, 6, 9, 10, 12, 13, 14, 15, and 16, as well as for mean PFS-16 score.

Because there was a positive correlation between PFS-16 score and worsening H&Y stage, we divided subjects into the H&Y stable group and the H&Y severely aggravated group, as described previously. The details of these two groups are shown in Table 3. There was no difference between the two groups in age, sex, disease duration, LEDD, and follow-up period. However, we found a significant difference in the initial H&Y stage and the H&Y stage during the follow-up period. The initial H&Y stage was found to be lower in the H&Y aggravated group compared with the H&Y stable group. Table 4 shows the comparisons of each question score in PFS-16 between these two groups. Compared with the H&Y stable group, scores were significantly higher in the H&Y aggravated group for questions 2, 4, 7, 9, 10, 12, 13, 14, and 16, as well as for the mean score. Among these nine increased scores, the *p* value was below 0.001 for questions 14 and 16.

## Discussion

In the present study, 62% of patients with PD were found to experience fatigue. As previously reported, fatigue is a common nonmotor symptom in PD patients.^13^ Fatigue was observed regardless of disease duration and LEDD. In addition, our study revealed a positive correlation between mean PFS-16 score and age or H&Y stage. In contrast, a previous study in Polish PD patients indicated that the prevalence of fatigue is independent of age but correlated with LEDD.^14^ However, a study of older adults in community living suggested that the incidence of fatigue is dependent on individuals aged 75 to 84.^15^ A further study with a larger cohort is needed to clarify these discrepancies.

Next, we found that the mean PFS-16 score and many individual PFS-16 scores were correlated with H&Y aggravation during the follow-up period. Although it is not clear whether disease severity in PD correlates with the prevalence of fatigue, Stocchi et al. reported that distressing fatigue is associated with higher total Unified Parkinson’s Disease Rating Scale (UPDRS) scores and poorer quality of life in Italian PD patients.^6^ Moreover, another study also indicated that fatigue severity increases with disease progression,^6^ and a further study reported that fatigue actually develops early in the disease, perhaps even in pre-motor periods.^16^ Based on these previous reports and our present results, fatigue severity could therefore be helpful in assessing PD prognosis. It may be useful to carefully observe the worsening of symptoms in patients with PD if they exhibit fatigue as per the PFS-16.

In comparisons between the H&Y stable group and the H&Y aggravated group, questions 14 and 16 showed the lowest *p* values compared with other questions. Because these two questions are related to fatigue when doing nothing, they represent a central fatigue, described as “the failure to initiate and/or sustain attentional tasks and physical activities requiring self-motivation”, rather than a peripheral fatigue.^17^ It is postulated that inflammatory processes might be involved in the development of pathological central fatigue,^18^ and accumulating evidence suggests an association between neuroinflammatory processes in the prodromal stage of PD and pathological progression in this disease.^19^ Therefore, questions 14 and 16 in the PFS-16 may represent ongoing neuroinflammatory processes in PD patients.

This study has some limitations. First, the sample size was relatively small, and the Brunner–Munzel test is more useful in a larger sample size. In addition, the differences between the H&Y stable group and the H&Y aggravated group, and the correlations between PFS-16 scores and H&Y aggravation severity, should be tested in a future larger cohort. Second, the follow-up period is rather short and varied between patients. Further investigations using a longer follow-up period are necessary to verify the present results.

## Figures and Tables

**Table1:** Correlation between the 16-item Parkinson Fatigue Scale score and change in Hoehn & Yahr stage (*n*=56)

Number	Question	Score (mean±SD)	Correlation with H&Y aggravation (*p* value/ρ)
Q-1	I have to rest during the day	3.51±1.22	0.184/
Q-2	My life is restricted by fatigue	3.25±1.31	0.035/0.28
Q-3	I get tired more quickly than other people I know	3.67±1.02	0.820/
Q-4	Fatigue is one of my three worst symptoms	3.42±1.21	0.054/
Q-5	I feel completely exhausted	3.33±1.08	0.041/0.27
Q-6	Fatigue makes me reluctant to socialise	3.33±1.23	0.010/0.33
Q-7	It takes me longer to get things done because of fatigue	3.62±1.12	0.050/
Q-8	I have a feeling of heaviness	3.41±1.10	0.423/
Q-9	If I wasn’t so tired I could do more things	3.50±1.17	0.003/0.38
Q-10	Everything I do is an effort	3.41±1.20	0.011/0.33
Q-11	I feel tired for much of the time	3.10±1.10	0.066/
Q-12	I feel totally drained	3.05±1.16	0.004/0.37
Q-13	Fatigue makes it difficult for me to cope with everyday activities	3.17±1.19	0.009/0.34
Q-14	I feel tired even when I haven’t done anything	2.98±1.15	0.009/0.34
Q-15	Because of fatigue I do less in my day than I would like	3.26±1.21	0.037/0.27
Q-16	I get so tired I want to lie down wherever I am	3.14±1.13	0.003/0.38
Mean		3.32±0.99	0.019/0.31

a) SD: standard deviation. H&Y: Hoehn & Yahr. ρ: Spearman’s rank correlation coefficient. b) In this table, we present the original version of PFS-16. We used the Japanese version of PFS-16 in the actual questionnaire.

**Table2:** Clinical features of subjects

Characteristic		Correlation with mean PFS-16 (*p* value/ρ)
Number of patients	56	—
Age; mean±SD (range, years)	72.5±7.45 (55–92)	0.029/0.29
Male/female	25/31	—
Duration; mean±SD (range, years)	6.39±4.90 (1–23)	0.128/
Levodopa equivalent daily dose mean±SD (mg)	406.32±229.39	0.392/
Initial H&Y stage; (median: mean±SD)	3: 3.09±0.91	0.043/0.27
Follow-up H&Y stage; (median: mean±SD)	4: 3.77±1.01	*p*<0.001/0.45
Follow-up period; mean±SD (range, month)	26.71±10.67 (2– –34)	0.083/
H&Y aggravation during follow-up period; mean±SD	0.67±0.70	0.019/0.31
Mean PFS-16 score, mean±SD	3.32±0.99	—

a) SD: standard deviation. H&Y: Hoehn and Yahr. PFS-16: The 16-item Parkinson Fatigue Scale. ρ: Spearman’s rank correlation coefficient.

**Table3:** Clinical features of Hoehn & Yahr stable and aggravated groups

Characteristic	H&Y stable group	H&Y aggravated group
Number of patients	26	7
Age; mean±SD (range, years)	70.8±7.23 (55–83)	75.2±6.92 (68–84)
Male/female	14/12	3/4
Duration; mean±SD (range, years)	6.76±5.57 (1–23)	4.14±3.28 (1–9)
Levodopa equivalent daily dose (mean±SD (mg))	440.89±256.18	322.75±86.48
Initial H&Y stage; (median: mean±SD)	3: 3.19±0.98	2: 2.28±0.75*
Follow-up H&Y stage; (median: mean±SD)	3: 3.19±0.98	4: 4.28±0.75**
Follow-up period; (mean±SD (range, month))	29.03±9.95 (2–34)	18.28±11.16 (4–34)

a) SD: standard deviation. H&Y: Hoehn and Yahr. b) * *p*=0.011, ** *p*=0.002 c) Brunner–Munzel test was used for intergroup comparisons.

**Table4:** Comparison for the 16-item Parkinson Fatigue Scale between H&Y stable and aggravated groups

Number	H&Y stable group (*n*=26)	H&Y aggravated group (*n*=7)	*p* value
Q-1	3.23±1.45	4.00±0.81	0.181
Q-2	2.84±1.43	4.00±0.57	0.007
Q-3	3.69±1.08	4.14±0.69	0.369
Q-4	3.15±1.37	4.14±0.37	0.009
Q-5	3.07±1.16	3.85±0.69	0.055
Q-6	2.88±1.24	3.85±1.06	0.061
Q-7	3.30±1.28	4.14±0.37	0.029
Q-8	3.30±1.19	3.57±0.78	0.561
Q-9	3.07±1.26	4.28±0.75	0.009
Q-10	3.00±1.29	4.00±0.81	0.031
Q-11	2.80±1.13	3.28±1.11	0.306
Q-12	2.61±1.16	3.71±0.75	0.003
Q-13	2.73±1.31	3.71±0.75	0.012
Q-14	2.61±1.23	3.85±0.37	<0.001
Q-15	2.88±1.39	3.71±0.95	0.092
Q-16	2.73±1.15	3.85±0.69	<0.001
mean	2.99±1.08	3.88±0.49	0.012

a) H&Y: Hoehen and Yahr. b) Brunner-Munzel test was used for inter-group comparison.
